# Phenotypic and Genomic Characterization of Vancomycin Non-Susceptibility in Multidrug-Resistant *Enterococcus* spp. From Hungarian Poultry

**DOI:** 10.3390/antibiotics15020131

**Published:** 2026-01-28

**Authors:** Ádám Kerek, Gergely Tornyos, Krisztián Bányai, Eszter Kaszab, Ákos Jerzsele

**Affiliations:** 1 Department of Pharmacology and Toxicology, University of Veterinary Medicine, István utca 2, H-1078 Budapest, Hungary; tornyos.gergely.almos@student.univet.hu (G.T.); banyai.krisztian@univet.hu (K.B.); jerzsele.akos@univet.hu (Á.J.); 2 National Laboratory of Infectious Animal Diseases, Antimicrobial Resistance, Veterinary Public Health and Food Chain Safety, University of Veterinary Medicine, István utca 2, H-1078 Budapest, Hungary; kaszab.eszter@univet.hu; 3 Department of Medical Biology, Medical School, University of Pécs, H-7624 Pécs, Hungary; 4 HUN-REN Veterinary Medical Research Institute, H-1143 Budapest, Hungary; 5 One Health Institute, University of Debrecen, Nagyerdei krt. 98, H-4032 Debrecen, Hungary; 6 Department of Microbiology and Infectious Diseases, University of Veterinary Medicine, István utca 2, H-1078 Budapest, Hungary

**Keywords:** *Enterococcus* spp., poultry, antimicrobial resistance, minimum inhibitory concentration, vancomycin, whole-genome sequencing, one health

## Abstract

**Background**: Vancomycin is a critically important antimicrobial in human medicine, and vancomycin-non-susceptible enterococci represent a One Health concern when animal reservoirs contribute to the wider resistance ecology. We aimed to characterize vancomycin non-susceptibility among poultry-derived *Enterococcus* spp. from Hungary, using a combined phenotypic–genomic approach. **Methods**: Following a phenotypic pre-screen with antimicrobials authorized for poultry, 218 isolates with elevated minimum inhibitory concentrations (MICs) were selected for extended broth microdilution testing including vancomycin. Vancomycin susceptibility was interpreted using Clinical and Laboratory Standards Institute (CLSI) clinical breakpoints and European Committee on Antimicrobial Susceptibility Testing (EUCAST) epidemiological cut-off values (ECOFFs). Whole-genome sequencing was performed on a targeted multidrug resistant (MDR) subset (*n* = 42), enriched for elevated or borderline vancomycin MICs and stratified by region and host species (chicken, turkey), and resistance determinants were annotated against the Comprehensive Antibiotic Resistance Database (CARD) using stringent similarity/coverage thresholds. **Results**: Among the 218 pre-screened isolates (126 from chickens; 92 from turkeys), 196 (89.9%) met MDR criteria. For vancomycin, 15.6% of isolates were resistant and 9.2% intermediate by CLSI, while EUCAST ECOFF classification placed 34.9% in the non-wild-type group. The vancomycin MIC distribution was right shifted, with high-end MICs observed. In the sequenced subset, vancomycin-associated determinants consistent with the *vanC* pathway (including regulatory and auxiliary components) were detected in five isolates. Beyond vancomycin-related determinants, the WGS subset harbored common resistance genes consistent with the observed multidrug-resistant phenotypes. **Conclusions**: Vancomycin non-susceptibility was detected among pre-screened poultry-derived *Enterococcus* isolates in Hungary, and genomic analysis revealed *vanC*-associated and other peptide antibiotic resistance signatures. These findings support targeted One Health surveillance integrating MIC distributions with genomic resistance determinants in food animal reservoirs.

## 1. Introduction

Antimicrobial resistance (AMR) is among the most pressing challenges to animal and public health in the 21st century, driven by interconnected selective pressures across clinical medicine, animal production, and the environment within a One Health continuum [1]. Globally, the scale of antimicrobial use in animals remains substantial, and international monitoring frameworks (WOAH—World Organization for Animal Health) highlight that veterinary-sector consumption represents a major component of the overall selection landscape [2,3]. Beyond direct therapeutic failure, AMR imposes a measurable societal burden, including excess mortality and increased healthcare costs [4,5,6].

Enterococci occupy a unique position in this context. As ubiquitous members of the gastrointestinal microbiota of humans and animals, *Enterococcus* spp. combine ecological persistence with a notable capacity to acquire and disseminate resistance determinants, which underpins their importance in healthcare-associated infections [7,8,9]. Clinically, *Enterococcus faecium* is widely recognized among the key multidrug-resistant pathogens of nosocomial relevance, and vancomycin-resistant *E. faecium* is repeatedly prioritized in global agendas for antibiotic research and development due to limited therapeutic alternatives [10]. At the population level, exposure to broad-spectrum antimicrobials can promote intestinal overgrowth and prolonged carriage, facilitating transmission within healthcare systems and between reservoirs [7,8,9,11].

From a veterinary and food-safety perspective, poultry production represents a high-throughput ecological niche where enterococci are common commensals but can act as opportunistic pathogens under predisposing conditions. In broilers and other poultry species, *E. faecalis*, *E. faecium*, *E. cecorum* and related species have been associated with clinically relevant syndromes including vertebral osteomyelitis/spondylitis, arthritis/tenosynovitis, osteomyelitis, and endocarditis, with particular susceptibility during the early post-hatch period [12,13,14,15,16]. Importantly, food-producing animals can also serve as reservoirs of resistant enterococci and resistance genes, creating potential pathways for dissemination via the food chain, occupational exposure, and environmental routes—an archetypal One Health interface [1,17,18,19].

Vancomycin is a glycopeptide antimicrobial of critical importance in human medicine, and glycopeptides are classified as highly prioritized medically important antimicrobials in World Health Organization (WHO) frameworks [20]. Mechanistically, vancomycin inhibits cell-wall synthesis by binding the D-Ala-D-Ala termini of peptidoglycan precursors, and susceptibility categories are routinely defined using standardized minimum inhibitory concentration (MIC)-based criteria [21,22,23]. While glycopeptides are not used in food-producing animals in the European Union, historical selection pressures are highly relevant: the glycopeptide growth promoter avoparcin—structurally related to vancomycin—was banned in the EU in 1997 due to concerns about selecting vancomycin-resistant enterococci (VRE) in livestock, and multiple studies documented persistence of VRE in poultry production systems even after the ban [24]. More recently, European Union-wide regulatory measures have further tightened the framework for veterinary antimicrobial stewardship (e.g., Regulation (EU) 2019/6 and subsequent implementing acts), reinforcing restrictions and the “human reserve” concept for certain antimicrobials [3,25]. Together, these developments highlight a central surveillance challenge: even in settings where direct glycopeptide exposure is absent, resistant enterococci and glycopeptide resistance determinants may persist and circulate due to co-selection, ecological stability, and transmission across compartments [1,24,26].

Vancomycin non-susceptibility in enterococci is mechanistically heterogeneous. High-level, acquired glycopeptide resistance is classically associated with transferable van gene clusters (e.g., *vanA*/*vanB*), while intrinsic low-level resistance mediated by *vanC* is typically linked to specific species groups (e.g., *E. gallinarum* and *E. casseliflavus* or *E. flavescens*) [27]. In this context, integrating phenotypic MIC distributions (including epidemiological cut-off-based wild-type/non-wild-type classification where applicable) with genomic resistance determinants provides a more informative and reviewer-proof framework than breakpoint-only reporting, particularly for ecology-focused One Health studies [21,22].

Building on susceptibility surveillance in poultry-associated enterococci, the present work adds a targeted vancomycin-centric layer by integrating isolate-level whole-genome sequencing (WGS) resistome profiling with phenotypic MIC data to inform One Health interpretation of glycopeptide-associated mechanisms in a food animal reservoir [28,29]. Although European poultry AMR surveillance has reported *Enterococcus* resistance patterns—typically emphasizing macrolides and tetracyclines—comparative data specifically addressing vancomycin non-susceptibility in poultry together with genomic context remain limited, particularly from Central and Eastern Europe. By combining a large-scale phenotypic screen with an MIC-enriched subset and targeted whole-genome sequencing, our study adds region-specific, One Health-relevant evidence on vancomycin-associated resistance signatures in poultry-derived *Enterococcus* spp. [30,31].

Against this background, the present study aimed to characterize vancomycin non-susceptibility among multidrug-resistant *Enterococcus* spp. isolated from Hungarian poultry by combining standardized broth microdilution MIC testing with genomic annotation of resistance determinants. By aligning phenotypic MIC shifts with vancomycin-associated genetic signatures, we sought to generate actionable evidence for targeted One Health surveillance in a food animal reservoir.

## 2. Results

### 2.1. Antibacterial Panel and Enterococcus Isolates Included in the Analyses

A total of 969 *Enterococcus* spp. isolates originating from Hungarian poultry production were included from a previously established strain collection and screened phenotypically. No animals were sampled or handled specifically for the present study; all analyses were performed exclusively on archived bacterial isolates. In the primary broth microdilution panel, isolates were tested against amoxicillin, neomycin, oxytetracycline, doxycycline, florfenicol, tylosin, tilmicosin, lincomycin, tiamulin, colistin, enrofloxacin, and trimethoprim–sulfamethoxazole.

Following this primary screen, 218 isolates (126 from chickens and 92 from turkeys) were selected for extended broth microdilution testing based on elevated MIC values to one or more compounds in the primary panel, prioritizing isolates categorized as resistant where CLSI interpretive criteria were available and isolates at the upper end of the tested MIC range for compounds lacking validated breakpoints. This study therefore focuses on resistance patterns within a phenotypically enriched collection rather than estimating the on-farm prevalence of resistance among all poultry-derived enterococci. The extended panel included amoxicillin–clavulanic acid, imipenem, ceftriaxone, spectinomycin, azithromycin, and vancomycin.

Species identification by Matrix-Assisted Laser Desorption/Ionization—Time-of-Flight Mass Spectrometry (MALDI-TOF MS) showed that the 218 isolates comprised predominantly *E. faecalis* (*n* = 114), *E. faecium* (*n* = 66), *E. gallinarum* (*n* = 16), and other *Enterococcus* species (*n* = 22). Vancomycin non-susceptibility (CLSI) was mainly observed in *Enterococcus faecium*, whereas isolates carrying *vanC*-associated determinants were confined to *E. gallinarum*.

### 2.2. Susceptibility to Antimicrobials Authorised for Poultry Use

Among the 18 tested agents, 12 are authorized for use in poultry and were therefore prioritized for interpreting field-relevant susceptibility patterns (amoxicillin, neomycin, oxytetracycline, doxycycline, tylosin, tilmicosin, lincomycin, tiamulin, florfenicol, enrofloxacin, colistin, and trimethoprim–sulfamethoxazole). Susceptibility results are summarized in Table 1, and MIC distributions are shown in Supplementary Figures S1 and S2.

For amoxicillin, 67.0% of isolates were susceptible and 33.0% resistant (MIC_90_ = 512 µg/mL). For oxytetracycline, 28.9% were susceptible, 1.8% intermediate, and 69.3% resistant (MIC_90_ = 256 µg/mL). For doxycycline, 34.4% were susceptible, 12.8% intermediate, and 52.8% resistant (MIC_90_ = 64 µg/mL). For tylosin, 31.7% were susceptible, 0.9% intermediate, and 67.4% resistant (MIC_90_ 512 µg/mL). Lincomycin activity was limited: 1.4% of isolates were susceptible, 0.9% intermediate, and 97.7% resistant (MIC_90_ > 512 µg/mL). For florfenicol, 5.0% were susceptible, 34.4% intermediate, and 60.6% resistant (MIC_90_ = 64 µg/mL). For enrofloxacin, 45.9% were susceptible, 11.9% intermediate, and 42.2% resistant (MIC_90_ = 32 µg/mL).

For neomycin, tilmicosin, tiamulin, colistin, and trimethoprim–sulfamethoxazole, validated interpretive criteria (clinical breakpoints and/or epidemiological cut-off values (ECOFFs)) were not available within the applied framework; therefore, MIC distributions are reported descriptively without S/I/R categorization. Accordingly, elevated MIC values for these agents should not be interpreted as ‘acquired resistance’ in the clinical breakpoint sense.

### 2.3. Agents of Critical Relevance to Human Medicine Included in the Extended Panel

Six agents in the 18-drug panel are primarily used in human medicine and are not routinely indicated for poultry in standard veterinary practice: amoxicillin–clavulanic acid, imipenem, ceftriaxone, spectinomycin, azithromycin, and vancomycin. Results for these agents are summarized in Table 2, and MIC distributions are shown in Supplementary Figure S3.

For amoxicillin–clavulanic acid, 82.6% of isolates were susceptible, 3.7% intermediate, and 13.8% resistant (MIC_90_ = 32 µg/mL). For imipenem, 92.7% were susceptible and 7.3% resistant (MIC_90_ = 4 µg/mL). For azithromycin, 33.5% were susceptible, 4.1% intermediate, and 62.4% resistant (MIC_90_ = 128 µg/mL).

Vancomycin non-susceptibility was detected in the enriched subset: 75.2% of isolates were susceptible, 9.2% intermediate, and 15.6% resistant, with a high MIC_90_ value of 256 µg/mL. Ceftriaxone MICs were recorded for completeness but were not interpreted, as *Enterococcus* spp. are intrinsically non-susceptible to cephalosporins. For spectinomycin, validated interpretive criteria were not available within the applied framework; therefore, MIC values are reported descriptively (MIC_90_ > 512 µg/mL) without Susceptible/Intermediate/Resistant categorization.

### 2.4. EUCAST ECOFF-Based Wild-Type/Non-Wild-Type Classification

Using EUCAST ECOFFs [32] the 218 pre-screened *Enterococcus* spp. isolates were classified as wild-type (WT) or non-wild-type (NWT) for agents where ECOFFs were available. The proportions of NWT isolates were 45.4% for amoxicillin, 93.6% for doxycycline, 60.6% for florfenicol, 14.7% for imipenem, 80.3% for neomycin, 71.1% for oxytetracycline, and 34.9% for vancomycin (Table 3).

For agents where both clinical breakpoints (CLSI) and EUCAST ECOFFs were available, we compared clinical categorization (Susceptible/Intermediate/Resistant) with WT/NWT classification. Across these agents, ECOFF-based classification consistently assigned a higher proportion of isolates to the NWT group than the proportion classified as clinically resistant by CLSI (Table 4). This pattern is expected because ECOFFs delineate the upper end of the phenotypic WT MIC distribution and are intended primarily for detecting isolates with phenotypically detectable acquired resistance mechanisms (surveillance purpose), whereas clinical breakpoints are designed for guiding therapy and incorporate pharmacokinetic/pharmacodynamic (PK/PD) and clinical outcome considerations; therefore, WT/NWT status and clinical Susceptible/Intermediate/Resistant categories do not necessarily coincide

### 2.5. MDR, XDR and PDR Phenotypes

Across the 218 *Enterococcus* spp. isolates, multidrug resistance was evaluated using the nine antimicrobial classes represented in the poultry-authorized primary panel (penicillins, aminoglycosides, tetracyclines, macrolides, lincosamides, pleuromutilins, phenicols, fluoroquinolones, and folate pathway inhibitors). Multidrug resistance (MDR) was defined [33] as non-susceptibility to at least one agent in three or more antimicrobial classes. Based on this criterion, 196/218 isolates (89.9%) were classified as MDR.

Within the MDR subset, eight isolates remained susceptible to agents in no more than two antimicrobial classes and were therefore classified as putative extensively drug-resistant (XDR) within the scope of the tested panel. One isolate was non-susceptible to all agents included in our panel and was thus classified as putative pandrug-resistant (PDR) within the scope of the tested panel.

The breadth of resistance among MDR isolates was heterogeneous. Of the 196 MDR isolates, 34 (17.3%) were non-susceptible in three antimicrobial classes, 54 (27.5%) in four classes, 55 (28.1%) in five classes, 37 (18.9%) in six classes, 7 (3.6%) in seven classes, 8 (4.1%) in eight classes, and 1 (0.5%) in all nine classes (Table 5).

### 2.6. Regional Distribution of MDR Enterococcus Isolates

Among the 196 MDR *Enterococcus* spp. isolates, 15 (7.6%) originated from the Dél-Alföld, 22 (11.2%) from Észak-Magyarország, 25 (12.8%) from Nyugat-Dunántúl, 29 (14.8%) from Közép-Magyarország, 31 (15.8%) from the Észak-Alföld, 35 (17.9%) from Dél-Dunántúl, and 39 (19.9%) from Közép-Dunántúl. The distribution of MDR phenotypes across regions and resistance profiles by antimicrobial class is summarized in Table 6. The geographic delineation of the seven Hungarian NUTS-2 regions is provided in Supplementary Figure S4.

### 2.7. Sequenced Subset and Resistome Overview

Whole-genome sequencing was performed on a targeted subset of 42 MDR isolates from the phenotypically enriched collection, selected based on elevated vancomycin MIC values (stratified by region and host species as described in Methods). The WGS subset was used to describe resistance determinants associated with vancomycin and related peptide-antibiotic phenotypes and was not intended for population-level prevalence inference. Resistance determinants were annotated against Comprehensive Antibiotic Resistance Database (CARD) using Resistance Gene Identifier (RGI); only ‘strict’ (or higher) hits meeting high-stringency filters (≥95% sequence identity and ≥95% reference coverage) were interpreted in the main text. To avoid overinterpretation of spurious or partial matches, only curated, high-confidence determinants are summarized in the Results, while the complete hit-level output is provided in Supplementary Table S1.

### 2.8. Detection of Vancomycin-Associated Determinants

Among the 42 sequenced isolates, five carried gene signatures were annotated as vancomycin resistance determinants, including components of a *vanC*-associated module (*vanC* and selected regulatory/auxiliary elements). Across these five isolates, four showed co-detection of multiple *vanC*-associated components (*vanC* together with a combination of *vanR*/*vanS*/*vanT*/*vanXY*), whereas one isolate carried *vanC* without the accompanying regulatory/auxiliary genes listed above (Table 7).

Isolate-level phenotypes for the five *vanC*-associated isolates are summarized in Table 7, including vancomycin MICs of 512 µg/mL (isolate 142; *E. gallinarum*), 512 µg/mL (isolate 198; *E. gallinarum*), 512 µg/mL (isolate 322; *E. gallinarum*), 4 µg/mL (isolate 432; *E. gallinarum*), and 256 µg/mL (isolate 444; *E. gallinarum*). Notably, isolate 432 had a vancomycin MIC equal to the ECOFF threshold (4 µg/mL), resulting in NWT classification by ECOFF despite being categorized as susceptible by CLSI breakpoints.

When mapped to vancomycin MIC values in the *vanC*-associated subset, four isolates carrying multi-component *vanC*-associated signatures displayed high vancomycin MICs (256–512 µg/mL), whereas the isolate with *vanC* detected in isolation showed a low vancomycin MIC (4 µg/mL).

### 2.9. Genomic Correlates for Vancomycin Non-Susceptibility in the WGS Subset

Whole-genome sequencing was performed on a targeted subset of 42 MDR isolates selected from the phenotypically enriched collection to enable genomic characterization of resistance determinants. The genomic results are reported exclusively in relation to vancomycin- and peptide antibiotic-associated resistance signatures.

In silico screening identified vancomycin-associated determinants consistent with a *vanC*-linked pathway in five isolates (Table 7). Four isolates carried *vanC* together with multiple auxiliary/regulatory components (*vanR*/*vanS*/*vanT*/*vanXY*), whereas one isolate had *vanC* detected without these additional components.

No acquired high-level glycopeptide resistance gene clusters were detected in the WGS subset: *vanA* and *vanB* were absent in all 42 genomes. Within the five isolates carrying *vanC*-linked signatures, vancomycin MICs ranged from 4 to 512 µg/mL (Table 7), with four isolates showing high vancomycin MIC values (256–512 µg/mL).

## 3. Discussion

Harmonized antimicrobial resistance (AMR) monitoring in the European Union provides an essential benchmark for interpreting resistance trends in food-producing animals, including poultry, by combining phenotypic antimicrobial susceptibility testing (AST) with standardized interpretive frameworks [34]. This monitoring is underpinned by EU-level rules for harmonized AMR reporting and is coordinated through European Food Safety Authority/European Centre for Disease Prevention and Control (EFSA/ECDC) reporting streams [35]. Within such frameworks, poultry-associated *Enterococcus* spp. are relevant both as commensals that reflect antimicrobial selection pressure in production systems and as opportunistic pathogens with One Health relevance.

In addition to clinical breakpoints, EUCAST ECOFFs support the separation of WT populations from NWT isolates that show phenotypically detectable acquired resistance mechanisms [32]. Importantly, ECOFFs are not designed to predict clinical outcome; rather, they serve surveillance and resistance-detection purposes and can therefore classify isolates as NWT even when they remain clinically treatable under breakpoint-based criteria [32]. This conceptual distinction provides a coherent explanation for our observation that ECOFF-based NWT proportions exceeded CLSI-defined clinical resistance for antimicrobials with both interpretive systems available, delineating a “grey zone” of early or low-level phenotypic resistance signals that may not necessarily translate into treatment failure [32].

In the present work, the susceptibility of 218 *Enterococcus* spp. isolates was determined against 18 antimicrobials, following an initial screening of a larger isolate set and subsequent selection of a subset enriched for elevated MICs. This sampling strategy is critical for interpreting absolute resistance proportions: the resulting resistance and MDR frequencies are expected to be higher than those obtained from unselected, population-representative sampling and should be compared to external surveillance data with appropriate caution.

For poultry-authorized antimicrobials, resistance was particularly pronounced against lincosamides and tetracyclines. The near-universal resistance to lincomycin (97.7%) aligns with prior reports describing high MICs and high resistance proportions in poultry-associated enterococci, including similarly elevated MIC_90_ values [30]. Tetracycline-class resistance is consistently reported as high across geographic settings in poultry-associated enterococci [30,36,37,38,39], and our oxytetracycline and doxycycline findings fall within the upper range of published values, while also highlighting substantial between-country variability likely driven by antimicrobial usage patterns and local clonal composition [36,37,38,39]. Macrolide resistance (tylosin) likewise remained high and broadly concordant with earlier poultry-focused studies, while showing notable heterogeneity across datasets [30,38]. For fluoroquinolones, our enrofloxacin resistance level was moderate relative to several poultry reports describing substantially higher resistance [38,39], yet still indicates considerable selection pressure within the studied system [36,38,39].

Amoxicillin showed the most favorable phenotypic profile among poultry-authorized agents; nevertheless, the resistance proportion remains high in absolute terms and contrasts with reports describing near-complete ampicillin susceptibility in some poultry *Enterococcus* collections [30,36,40,41]. This discrepancy is plausibly explained by (i) our enriched sampling design targeting elevated MIC phenotypes, (ii) potential differences in species composition and clonal structure, and (iii) host- and context-specific applicability of interpretive criteria across studies. For several poultry-authorized agents (e.g., neomycin, tilmicosin, tiamulin, colistin, and potentiated sulfonamides), the lack of validated clinical breakpoints limits categorical interpretation; accordingly, MIC distributions and summary statistics (e.g., MIC_90_) are best treated as descriptive indicators of reduced susceptibility rather than direct proxies of clinical resistance.

Among antimicrobials primarily used in human medicine in our panel, amoxicillin–clavulanic acid and imipenem showed largely preserved activity, consistent with multiple reports describing low resistance levels for carbapenems in enterococci from animal-associated settings [40,41]. Azithromycin resistance remained high, which is compatible with broad macrolide resistance trends in enterococci and supports the view that macrolide/lincosamide selection can be sustained by antimicrobial classes used in production systems [30,38,39,41,42]. For ceftriaxone, high MICs are expected because enterococci are intrinsically resistant to cephalosporins as a drug class; therefore, ceftriaxone MIC distributions should be interpreted in the context of intrinsic non-susceptibility rather than acquired resistance [43]. For spectinomycin, the absence of well-established clinical breakpoints again warrants a descriptive interpretation; a high MIC_90_ suggests reduced susceptibility, but categorical resistance claims should be avoided without validated interpretive criteria.

Regional heterogeneity in MDR profiles may reflect differences in farm density, production intensity, and antimicrobial use practices, as well as local biosecurity and management patterns that influence transmission and selection pressure. Because the present dataset was enrichment-based and not designed for region-level causal inference, these factors are proposed as plausible contributors that warrant targeted, prospective One Health surveillance.

Vancomycin resistance in enterococci has high public health relevance, reflected by the World Health Organization’s prioritization of vancomycin-resistant *Enterococcus faecium* (VREfm) among the high-priority bacterial threats requiring intensified research and development and public health attention [44]. In our phenotypic dataset, 15.6% of isolates were classified as vancomycin-resistant by breakpoint-based interpretation, with a high vancomycin MIC_90_ (256 µg/mL). However, genome-resolved analysis of a sequenced subset (*n* = 42) did not detect the major acquired high-level resistance determinants *vanA* or *vanB*, and the vancomycin-associated genotypic signals observed were dominated by *vanC*-linked signatures in five isolates.

This genetic pattern is biologically coherent: *vanC* is a chromosomally encoded, typically non-transferable determinant characteristic of *Enterococcus gallinarum* and *E. casseliflavus*, conferring intrinsic low-level vancomycin non-susceptibility [27]. Accordingly, the absence of *vanA/vanB* in the sequenced subset is reassuring with respect to the presence of classical acquired high-level VRE mechanisms in the analyzed genomes, and it suggests that at least part of vancomycin non-susceptibility signal in our study is attributable to species-associated intrinsic mechanisms rather than horizontally acquired *vanA/vanB* cassettes. Nevertheless, continued vigilance is warranted because *E. gallinarum*—despite its intrinsic *vanC* background—has been reported to acquire *vanA* in rare circumstances, indicating that poultry-associated enterococcal populations can, under suitable conditions, serve as platforms for the emergence and dissemination of clinically relevant glycopeptide resistance [45].

Notably, the highest vancomycin MICs observed in some *E. gallinarum* isolates exceed ranges commonly attributed to intrinsic *vanC*-associated phenotypes; therefore, we refrain from mechanistic attribution beyond the detected *vanC*-linked signatures within the applied high-stringency in silico framework and highlight this as a priority for targeted follow-up (e.g., expanded genotyping and confirmatory analyses).

Given the One Health implications, our results support the value of integrated phenotypic–genotypic surveillance approaches that (i) report MIC distributions transparently, (ii) interpret data within the constraints of available clinical breakpoints and ECOFF frameworks, and (iii) use genome-resolved methods to distinguish intrinsic from acquired vancomycin resistance mechanisms.

Population-genomic studies indicate that hospital-adapted *E. faecium* lineages (often termed clade A1) are evolutionarily related to a broader clade A population that includes many animal-associated strains (often termed clade A2), and occasional overlap of sequence types across poultry and clinical settings has been reported. Accordingly, without implying direct transmission, our findings align with a One Health perspective in which shared (often mobile) resistance determinants may circulate across reservoirs and therefore warrant integrated surveillance [46,47].

A major finding of this work is the high MDR frequency (89.9%) within the enriched isolate subset, including isolates compatible with XDR and PDR phenotypes under the studied antimicrobial-class panel [33]. While the enrichment strategy precludes direct extrapolation to national prevalence, the data clearly demonstrates that heavily resistant *Enterococcus* populations exist in poultry-associated reservoirs and can be captured by targeted sampling. This reinforces the need for structured, routine monitoring that combines representative surveillance sampling with risk-based targeted sampling to detect emerging resistance phenotypes and genotypes.

Key limitations should be explicitly acknowledged. First, for several antimicrobials relevant to poultry practice, validated poultry- and species-specific clinical breakpoints are unavailable, constraining categorical interpretation and necessitating reliance on available CLSI clinical criteria and/or descriptive MIC reporting [30,36,37,48,49,50,51]. Second, the WGS subset (*n* = 42) provides mechanistic insight but does not fully resolve the genetic basis of vancomycin phenotypes across the entire phenotype collection; thus, the absence of *vanA*/*vanB* should be interpreted strictly within the sequenced subset. Third, without detailed farm-level antimicrobial usage metadata and fine-scale clonal/phylogenomic resolution across all isolates, attributing resistance differences to specific drivers remains outside the scope of the present manuscript.

A key limitation is the intentional phenotypic enrichment of the analyzed collection: the 218 isolates were selected from a larger set of 969 poultry-derived isolates based on elevated MIC values in an initial screen. Consequently, the proportions reported here should be interpreted as resistance burdens within an enriched subset and should not be extrapolated as population-level prevalence estimates.

Despite these constraints, the study delivers a coherent, surveillance-relevant message: poultry-associated enterococci can display high resistance burdens to multiple antimicrobial classes, and vancomycin non-susceptibility signals in the analyzed genomes were primarily associated with intrinsic *vanC* backgrounds rather than acquired *vanA/vanB* determinants. Building on these findings, a logical next step is a national-scale, regionally resolved “gene map” of vancomycin resistance determinants coupled to standardized AST and genome-resolved surveillance, providing actionable One Health intelligence for stewardship and risk mitigation.

## 4. Materials and Methods

### 4.1. Origin of Samples and Isolate Collection

Sampling had been performed previously on clinically healthy, large-scale commercial chicken and turkey farms across the seven Hungarian NUTS-2 regions as part of routine field monitoring, aiming for broad geographic coverage. In each region, three chicken farms and three turkey farms were included; within farms, cloacal and tracheal swabs (15 and 15 per farm, respectively) were collected using sterile transport swabs. *Enterococcus* spp. isolates were recovered using standard bacteriological procedures, including selective plating on *Enterococcus* modified agar (Merck KGaA, Darmstadt, Germany) according to the manufacturer’s instructions; presumptive *Enterococcus* colonies (light pink to red) were subcultured to purity prior to downstream identification. Pure cultures were archived in the Microbank™ system (Pro-Lab Diagnostics, Richmond Hill, ON, Canada) and stored at −80 °C until further analysis. The present study utilized only these archived isolates; no study-specific animal sampling or experimental procedures were conducted. At the start of the present analyses, the strain collection comprised a total of 969 *Enterococcus* spp. isolates.

Presumptive enterococci were identified to the species level by a MALDI-TOF MS (Flextra-LAB Kft., Budapest, Hungary), using the manufacturer’s standard workflow. Briefly, colonies from fresh culture were applied to the target plate, overlaid with matrix solution, and spectra were acquired and matched against the reference library (Bruker Daltonics, MBT Compass Library DB-12827; Bremen, Germany). Identification was accepted at the manufacturer-recommended score thresholds for species-level assignment.

### 4.2. Antimicrobials and Preparation of Stock Solutions

Isolates were initially screened against antimicrobials commonly used in poultry production: amoxicillin, neomycin, oxytetracycline, doxycycline, florfenicol, tylosin, tilmicosin, lincomycin, tiamulin, colistin, enrofloxacin, and trimethoprim–sulfamethoxazole. Based on this screening, 218 isolates showing elevated MIC values were selected for extended testing. Colistin MICs are reported for completeness only; *Enterococcus* spp. are intrinsically non-susceptible to polymyxins. As a second panel, the selected isolates were tested against antimicrobials of primary relevance to human medicine: amoxicillin–clavulanic acid, ceftriaxone, imipenem, spectinomycin, azithromycin, and vancomycin.

Antimicrobial powders were obtained from Merck KGaA (Darmstadt, Germany). Stock solutions were prepared in accordance with CLSI recommendations [52], using the recommended solvents: amoxicillin–clavulanic acid and imipenem were dissolved in 0.1 mol/L phosphate buffer (pH 6.0); azithromycin and tilmicosin were pre-dissolved with ethanol to facilitate solubilization and subsequently diluted with distilled water; and all other compounds were dissolved in distilled water. Stock concentrations were 1024 µg/mL and were corrected for manufacturer-reported purity. Amoxicillin–clavulanic acid was tested at a fixed 2:1 ratio (amoxicillin: clavulanic acid). Trimethoprim/sulfamethoxazole was tested at a fixed 1:19 ratio (trimethoprim: sulfamethoxazole), in line with standard broth microdilution conventions.

### 4.3. Minimum Inhibitory Concentration (MIC) Determination

Phenotypic antimicrobial susceptibility was assessed by broth microdilution following CLSI methodology [52]. Frozen isolates were revived from Microbank stocks and prepared for testing using overnight cultures. Broth microdilution was performed in sterile 96-well microtiter plates (VWR International, LLC., Debrecen, Hungary) using cation-adjuvated Mueller–Hinton broth (CAMHB). Two-fold serial dilutions were prepared for each antimicrobial to cover the full measurement range applied in the study (two-fold dilution series; upper and lower limits defined per antimicrobial based on expected MIC distributions). The final test volume was 100 µL per well (90 µL antimicrobial dilution + 10 µL bacterial inoculum).

Inoculate were prepared by adjusting bacterial suspensions to 0.5 McFarland using a nephelometer (Thermo Fisher Scientific, Budapest, Hungary), followed by dilution to achieve a final inoculum of approximately 5 × 10^5^ CFU/mL in the test wells [52]. Growth control wells (inoculum without antimicrobial) and sterility control wells (broth only) were included on each plate. Plates were incubated at 35 ± 2 °C for 18–24 h. MICs were defined as the lowest antimicrobial concentration preventing visible growth and were read using the Sensititre SWIN automatic MIC reader (Thermo Fisher Scientific, Budapest, Hungary) with the VIZION system software v3.4 (Thermo Fisher Scientific, Budapest, Hungary, 2024).

Quality control (QC) was performed using *Enterococcus faecalis* ATCC 29212, and QC results were accepted only when MIC values fell within CLSI-recommended QC ranges.

### 4.4. Interpretation of Susceptibility and Definition of Resistance Categories

Where available, CLSI clinical breakpoints were used to categorize isolates as Susceptible, Intermediate, or Resistant [30,36,37,48,49,50,51]. Because poultry-specific clinical breakpoints for *Enterococcus* spp. are not available for several antimicrobial–host combinations, human *Enterococcus* spp. breakpoints were applied, consistent with common practice in comparable studies.

In parallel, EUCAST ECOFFs were used to classify isolates as WT or NWT (i.e., with phenotypically detectable acquired resistance) where ECOFFs were available [32]. For antimicrobials lacking validated clinical breakpoints and/or ECOFFs, results are presented as MIC distributions without categorical interpretation. This applied in cases where no species-specific, validated interpretive criteria exist for *Enterococcus* spp. (and/or for poultry isolates) and/or where intrinsic non-susceptibility limits the clinical meaning of categorical S/I/R assignments. Interpretive criteria were applied using CLSI VET06 (1st edition, 2017) for clinical categorization where applicable [53], while EUCAST ECOFF-based WT/NWT classification was derived from the MIC EUCAST database [54].

MDR was defined as resistance to at least one agent in three or more antimicrobial classes; XDR and PDR categories were interpreted within the constraints of the antimicrobial-class panel tested and are therefore reported as putative when full-class coverage was not available [33].

### 4.5. Whole-Genome Sequencing and Bioinformatic Analysis

Forty-two isolates were selected for whole-genome sequencing from the phenotypically enriched MDR collection based on elevated vancomycin MIC values, using a stratified design to cover geographic regions and poultry host species (*n* = 3 isolates per region; 7 regions × 3 = 21 per host species; total *n* = 42). Genomic DNA was extracted using the QIAamp DNA kit (Qiagen, Hilden, Germany) according to the manufacturer’s protocol. Paired-end Illumina sequencing was performed on a NextSeq platform [55].

Raw reads were quality-checked with FastQC v0.11.9 [56] and trimmed using TrimGalore v0.6.6 [57]. De novo assembly was performed using MEGAHIT v1.2.9 [58]. Open reading frames were predicted with Prodigal v2.6.3 [59], translated to protein sequences, and screened for antimicrobial resistance determinants using Resistance Gene Identifier (RGI) v5.1.0 against the CARD [60].

Only “strict” (or higher) matches were retained. ARG calls were filtered by sequence identity and coverage thresholds (≥95% for both). Hits below these thresholds were retained only in the complete per-isolate RGI export (Supplementary Table S1) but were not interpreted as evidence of gene presence in the main text. The presence/absence of vancomycin resistance determinants (including *vanA*, *vanB*, and *vanC* ligase genes) was assessed within this framework.

## 5. Conclusions

This study provides an integrated phenotypic and genome-resolved overview of antimicrobial susceptibility in poultry-associated *Enterococcus* spp. from Hungary. In an MIC-enriched subset (*n* = 218), resistance was frequent across multiple antimicrobial classes, resulting in a high MDR burden, underscoring the need for sustained stewardship and structured surveillance in the poultry sector. Vancomycin non-susceptibility was detected phenotypically, while genome analysis of a sequenced subset (*n* = 42) did not identify the canonical acquired high-level glycopeptide resistance determinants (*vanA*/*vanB*); instead, vancomycin-associated signatures were confined to a small number of *vanC*-linked isolates consistent with intrinsic species-associated backgrounds. Taken together, these findings highlight poultry-associated enterococci as a relevant One Health reservoir of multidrug resistance and support the value of combining MIC distributions, breakpoint/ECOFF-based interpretation, and WGS to distinguish intrinsic from acquired resistance mechanisms and inform risk-based monitoring strategies.

## Figures and Tables

**Table 1:** Distribution of minimum inhibitory concentration (MIC) values of the investigated *Enterococcus* isolates for antimicrobials authorised for poultry, including MIC_50_ and MIC_90_. For each antimicrobial, the upper row shows the number of isolates and the lower row shows the percentage distribution. Red vertical lines indicate the clinical breakpoint where available; agents lacking validated breakpoints are reported descriptively without categorical interpretation.

Antibiotics	Breakpoint	1024	512	256	128	64	32	16	8	4	2	1	0.5	0.25	0.125	0.06	0.03	0.015	≤0.015	MIC_50_	MIC_90_
	µg/mL																				
Amoxicillin	≥16	13	17	23	7	5	6	1	10	17	35	38	31	9	1	0	1	2	2	2	512
		6.0%	7.8%	10.6%	3.2%	2.3%	2.8%	0.5%	4.6%	7.8%	16.1%	17.4%	14.2%	4.1%	0.5%	0.0%	0.5%	0.9%	0.9%		
Neomycin	-	57	29	27	37	25	23	5	7	5	2	1								256	>512
		26.1%	13.3%	12.4%	17.0%	11.5%	10.6%	2.3%	3.2%	2.3%	0.9%	0.5%									
Oxytetracycline	≥16	2	7	35	49	25	19	14	4	6	15	18	17	5	1	1				64	256
		0.9%	3.2%	16.1%	22.5%	11.5%	8.7%	6.4%	1.8%	2.8%	6.9%	8.3%	7.8%	2.3%	0.5%	0.5%					
Doxycycline	≥16		1	1	6	25	33	49	28	28	14	17	2	6	4	1	1	1	1	16	64
			0.5%	0.5%	2.8%	11.5%	15.1%	22.5%	12.8%	12.8%	6.4%	7.8%	0.9%	2.8%	1.8%	0.5%	0.5%	0.5%	0.5%		
Tylosin	≥32	73	44	19	9	1	1	2	7	13	23	22	4							512	>512
		33.5%	20.2%	8.7%	4.1%	0.5%	0.5%	0.9%	3.2%	6.0%	10.6%	10.1%	1.8%								
Tilmicosin	-	45	11	20	17	13	16	13	4	31	36	5	4	0	0	0	0	2	1	32	>512
		20.6%	5.0%	9.2%	7.8%	6.0%	7.3%	6.0%	1.8%	14.2%	16.5%	2.3%	1.8%	0.0%	0.0%	0.0%	0.0%	0.9%	0.5%		
Lincomycin	≥8	85	42	12	25	32	4	10	2	2	0	3	0	1						512	>512
		39.0%	19.3%	5.5%	11.5%	14.7%	1.8%	4.6%	0.9%	0.9%	0.0%	1.4%	0.0%	0.5%							
Tiamulin	-	33	60	54	49	13	4	1	1	0	0	1	1	1						256	>512
		15.1%	27.5%	24.8%	22.5%	6.0%	1.8%	0.5%	0.5%	0.0%	0.0%	0.5%	0.5%	0.5%							
Florfenicol	≥8		1	4	12	5	15	22	73	75	11									8	64
			0.5%	1.8%	5.5%	2.3%	6.9%	10.1%	33.5%	34.4%	5.0%										
Enrofloxacin	≥4	1	1	3	5	5	7	28	25	17	26	46	31	14	2	1	5	1		2	32
		0.5%	0.5%	1.4%	2.3%	2.3%	3.2%	12.8%	11.5%	7.8%	11.9%	21.1%	14.2%	6.4%	0.9%	0.5%	2.3%	0.5%			
Colistin	-	133	46	10	9	3	0	3	14											>512	>512
		61.0%	21.1%	4.6%	4.1%	1.4%	0.0%	1.4%	6.4%												
Trimethoprim—sulfamethoxazole	-	62	25	9	15	2	5	18	22	15	18	16	8	1	2					128	>512
		28.4%	11.5%	4.1%	6.9%	0.9%	2.3%	8.3%	10.1%	6.9%	8.3%	7.3%	3.7%	0.5%	0.9%						

**Table 2:** Distribution of minimum inhibitory concentration (MIC) values of the investigated *Enterococcus* isolates for antimicrobials authorised for human medicine, including MIC_50_ and MIC_90_. For each antimicrobial, the upper row shows the number of isolates and the lower row shows the percentage distribution. Agents lacking validated breakpoints were reported descriptively without categorical interpretation; ceftriaxone was not interpreted due to intrinsic non-susceptibility of *Enterococcus* spp. to cephalosporins.

Antibiotics	1024	512	256	128	64	32	16	8	4	2	1	0.5	0.25	0.125	0.06	0.03	0.015	≤0.015	MIC_50_	MIC_90_
	µg/mL																			
Amoxicillin-clavulanic acid *					9	21	8	35	31	36	43	20	6	0	2	2	2	3	2	32
					4.1%	9.6%	3.7%	16.1%	14.2%	16.5%	19.7%	9.2%	2.8%	0.0%	0.9%	0.9%	0.9%	1.4%		
Imipenem							4	12	16	43	70	47	11	1	0	3	9	2	1	4
							1.8%	5.5%	7.3%	19.7%	32.1%	21.6%	5.0%	0.5%	0.0%	1.4%	4.1%	0.9%		
Ceftriaxone	63	47	27	21	8	3	11	38											256	>512
	28.9%	21.6%	12.4%	9.6%	3.7%	1.4%	5.0%	17.4%												
Spectinomycin	32	26	41	40	58	18	3												128	>512
	14.7%	11.9%	18.8%	18.3%	26.6%	8.3%	1.4%													
Azithromycin			17	35	26	12	24	22	9	8	15	10	6	5	2	11	16		16	128
			7.8%	16.1%	11.9%	5.5%	11.0%	10.1%	4.1%	3.7%	6.9%	4.6%	2.8%	2.3%	0.9%	5.0%	7.3%			
Vancomycin		13	12	3	5	1	6	15	21	28	72	31	5	4	2				1	256
		6.0%	5.5%	1.4%	2.3%	0.5%	2.8%	6.9%	9.6%	12.8%	33.0%	14.2%	2.3%	1.8%	0.9%					

* fixed ratio 2:1 (amoxicillin:clavulanic acid).

**Table 3:** EUCAST epidemiological cut-off values (ECOFFs) for the investigated antimicrobials and the distribution of non-wild-type (NWT) isolates, reported as counts and percentages for each antimicrobial.

Antibiotics	ECOFF	NWT	
		No. of Isolates (*n*)	%
Amoxicillin	≥4	99	45.4
Doxycycline	≥0.5	204	93.6
Florfenicol	≥8	132	60.6
Imipenem	≥4	32	14.7
Neomycin	≥64	175	80.3
Oxytetracycline	≥8	155	71.1
Vancomycin	≥4	76	34.9

**Table 4:** Percentage of resistant (R) isolates according to Clinical Laboratory Standards Institute (CLSI) clinical breakpoints and percentage of non-wild-type (NWT) isolates according to European Committee on Antimicrobial Susceptibility Testing (EUCAST) epidemiological cut-off values (ECOFFs) for the investigated antimicrobials.

Antibiotics	R (%)	NWT (%)
Amoxicillin	33.0	45.4
Imipenem	7.3	14.7
Oxytetracycline	69.3	71.1
Doxycycline	52.8	93.6
Florfenicol	60.6	60.6
Vancomycin	15.6	34.9

**Table 5:** Distribution of the 196 multidrug-resistant (MDR) isolates by the number of antimicrobial classes to which resistance was observed, reported as counts and percentages.

Resistance to Antimicrobial Classes	Isolates (n)	Isolates (%)
MDR-3	34	17.3
MDR-4	54	27.5
MDR-5	55	28.1
MDR-6	37	18.9
MDR-7	7	3.6
MDR-8	8	4.1
MDR-9	1	0.5

**Table 6:** Percentage distribution of multidrug-resistant (MDR) *Enterococcus* spp. isolates across Hungarian regions (7/7), stratified by the number of antimicrobial classes to which resistance was observed.

		DA	ÉM	ND	KM	ÉA	DD	KD	All
	
MDR-3		1.0	4.6	3.1	3.6	2.1	0.0	3.1	17.5
MDR-4		1.0	5.6	4.1	3.6	6.1	4.1	3.1	27.6
MDR-5		1.5	0.5	4.1	4.6	3.6	7.1	6.6	28.0
MDR-6		2.0	0.5	1.0	3.1	2.0	4.6	5.6	18.8
MDR-7		1.0	0.0	0.5	0.0	0.5	0.0	1.5	3.5
MDR-8		1.0	0.0	0.0	0.0	1.5	1.5	0.0	4.0
MDR-9		0.0	0.0	0.0	0.0	0.0	0.5	0.0	0.5
All		7.5	11.2	12.8	14.8	15.8	17.9	19.9	100.0

DA—Dél-Alföld; ÉM—Észak-Magyarország; ND—Nyugat-Dunántúl; KM—Közép-Magyarország; ÉA—Észak-Alföld; DD—Dél-Dunántúl; KD—Közép-Dunántúl.

**Table 7:** Vancomycin-associated (*vanC*-pathway) determinants in sequenced isolates. Abbreviations: VAN, vancomycin; Clinical Laboratory Standards Institute (CLSI) category based on VAN minimum inhibitory concentration (MIC) (S ≤ 4 µg/mL; I 8–16 µg/mL; R ≥ 32 µg/mL); European Committee on Antimicrobial Susceptibility Testing (EUCAST) epidemiological cut-off value (ECOFF) class for VAN based on epidemiological cut-off values (ECOFF) ≥ 4 µg/mL (NWT) vs. <4 µg/mL (WT). Gene presence and absence are indicated by “+” and “–“, respectively.

Isolate ID	Species	*vanC*	*vanR*	*vanS*	*vanT*	*vanXY*	VAN MIC (µg/mL)	CLSI Category	EUCAST ECOFF Class
142	*E. gallinarum*	+	+	+	+	+	512	R	NWT
198	*E. gallinarum*	+	–	+	+	+	512	R	NWT
322	*E. gallinarum*	+	+	+	+	+	512	R	NWT
432	*E. gallinarum*	+	–	–	–	–	4	S	NWT
444	*E. gallinarum*	+	+	+	+	+	256	R	NWT

## Data Availability

Raw sequencing reads have been deposited in the NCBI Sequence Read Archive (SRA) under BioProject accession PRJNA1394645. The data presented in this study are available from the corresponding author upon reasonable request.

## Supplementary Materials

The following supporting information can be downloaded at: https://www.mdpi.com/article/10.3390/antibiotics15020131/s1. Table S1: Isolate-level presence/absence of antimicrobial resistance determinants identified by whole-genome sequencing in the sequenced *Enterococcus* subset, including vancomycin-associated (*van*) determinants. Figure S1: Minimum inhibitory concentration (MIC) distributions of poultry-derived *Enterococcus* spp. isolates for selected antimicrobials. Figure S2: Minimum inhibitory concentration (MIC) distributions of poultry-derived *Enterococcus* spp. isolates for additional antimicrobials. Figure S3: Minimum inhibitory concentration (MIC) distributions of poultry-derived *Enterococcus* spp. isolates for antimicrobials primarily used in human medicine, including vancomycin. Figure S4: Geographic outline of Hungary showing the seven NUTS-2 regions used for regional stratification in this study (Dél-Alföld, Észak-Magyarország, Nyugat-Dunántúl, Közép-Magyarország, Észak-Alföld, Dél-Dunántúl, and Közép-Dunántúl).
