# Supplementary material for: Phenotypic and Genomic Characterization of Vancomycin Non-Susceptibility in Multidrug-Resistant Enterococcus spp. From Hungarian Poultry
Source: Antibiotics (Basel). 2026 Jan 28;15(2):131. doi: 10.3390/antibiotics15020131 (PMC12937384; doi:10.3390/antibiotics15020131)
Supplement: Supplementary file 1 [file antibiotics-15-00131-s001.zip › Supplementary materials figures.pdf]

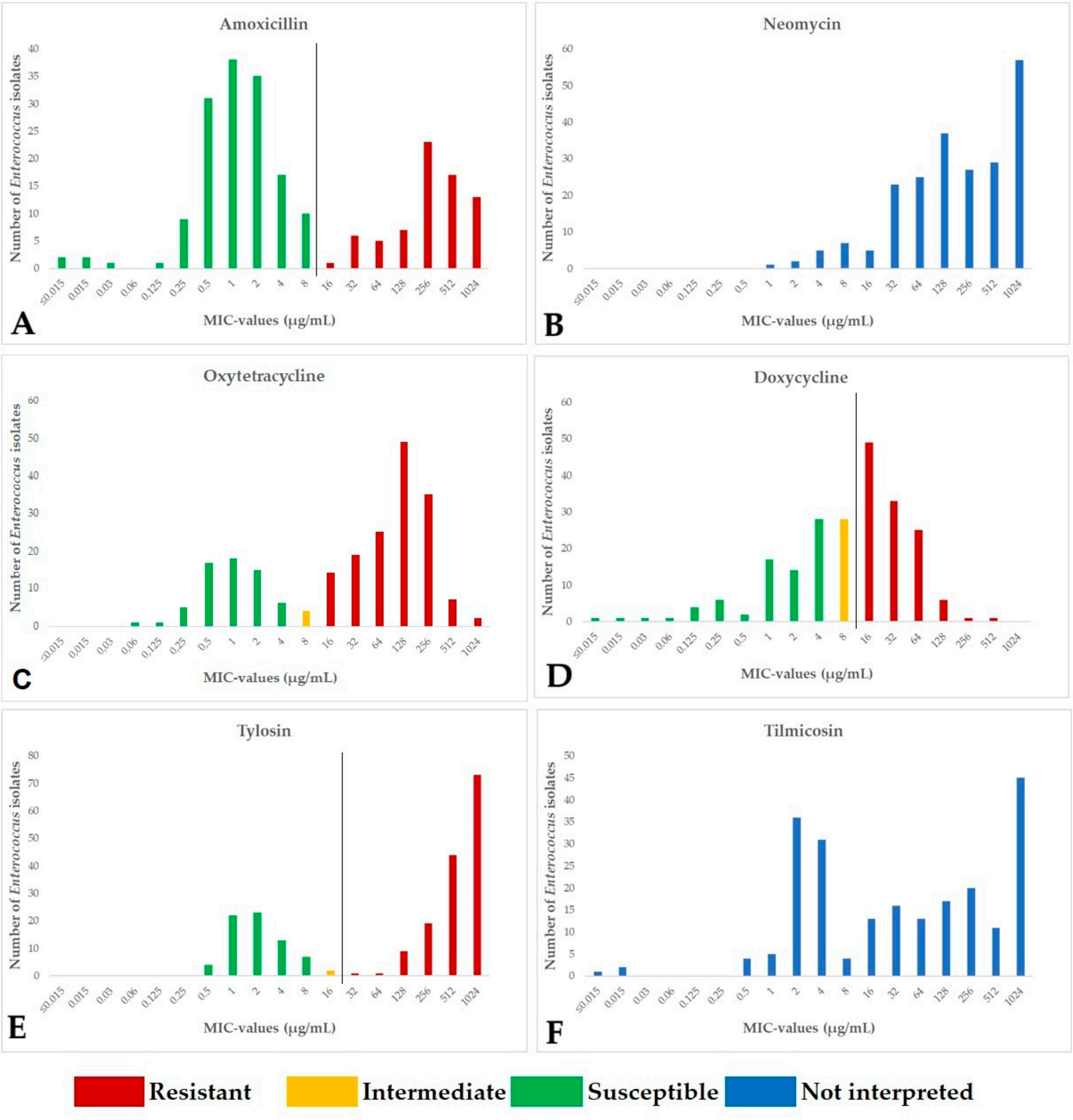

Supplementary Figure S1. Minimum inhibitory concentration (MIC) distributions of poultry-derived *Enterococcus* spp. isolates for selected antimicrobials. Bars represent the number of isolates at each broth microdilution concentration (two-fold dilution series; µg/mL). Colors denote Clinical Laboratory Standards Institute (CLSI) clinical categories (Susceptible/Intermediate/Resistant) where breakpoints are available; antimicrobials lacking clinical breakpoints are shown as Not interpreted (no clinical breakpoints available). Vertical dashed lines indicate clinical breakpoints. Panels: (A) amoxicillin, (B) neomycin, (C) oxytetracycline, (D) doxycycline, (E) tylosin, (F) tilmicosin. Intermediate category is displayed only for antimicrobials with defined CLSI intermediate breakpoints.

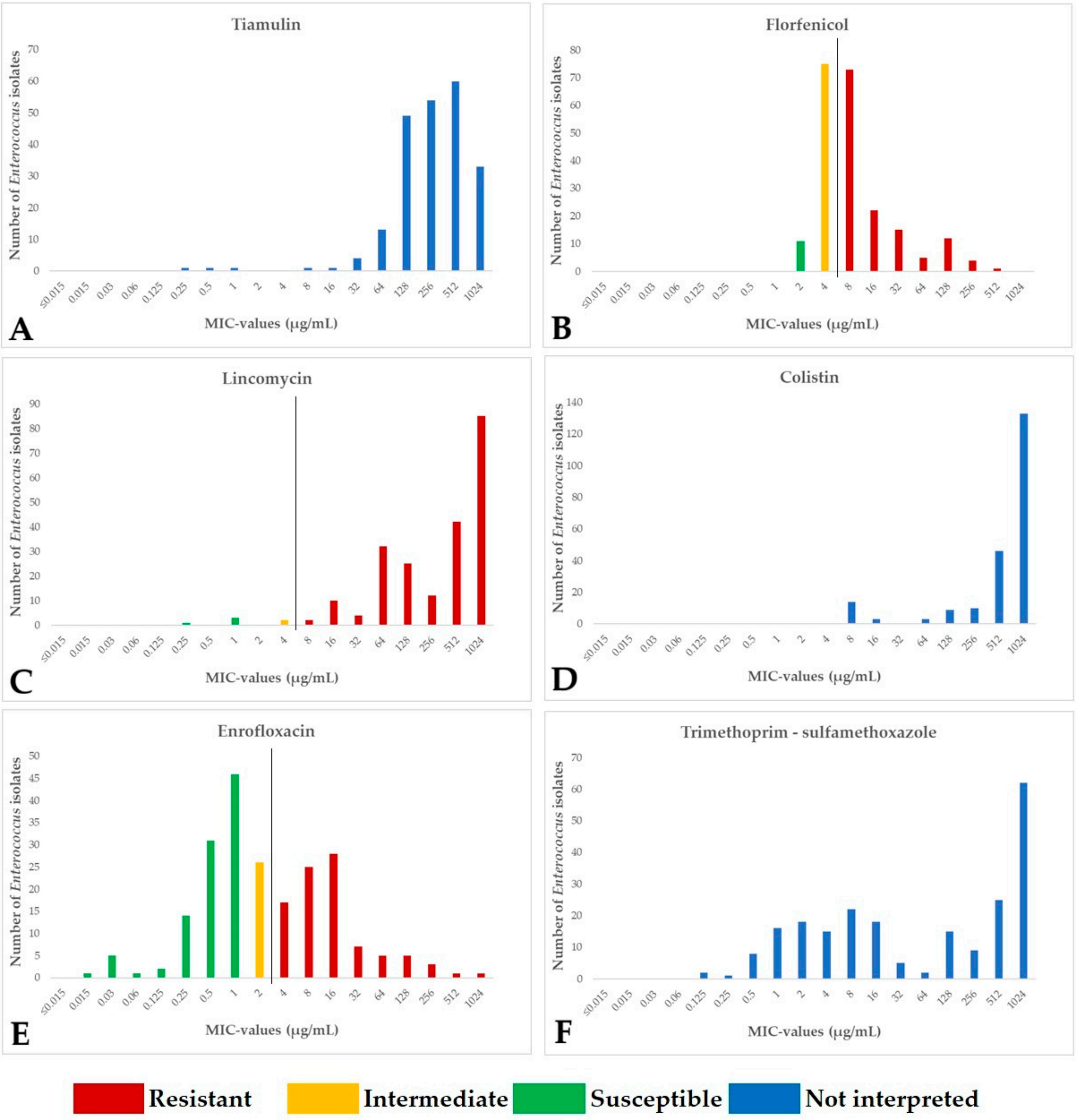

Supplementary Figure S2. Minimum inhibitory concentration (MIC) distributions of poultry-derived *Enterococcus* spp. isolates for additional antimicrobials. Bars represent the number of isolates at each broth microdilution concentration (two-fold dilution series; µg/mL). Colors denote Clinical Laboratory Standards Institute (CLSI) clinical categories (Susceptible/Intermediate/Resistant) where breakpoints are available; antimicrobials lacking clinical breakpoints are shown as Not interpreted (no clinical breakpoints available). Vertical dashed lines indicate clinical breakpoints. Panels: (A) tiamulin, (B) florfenicol, (C) lincomycin, (D) colistin, (E) enrofloxacin, (F) trimethoprim–sulfamethoxazole. Intermediate category is displayed only for antimicrobials with defined CLSI intermediate breakpoints.

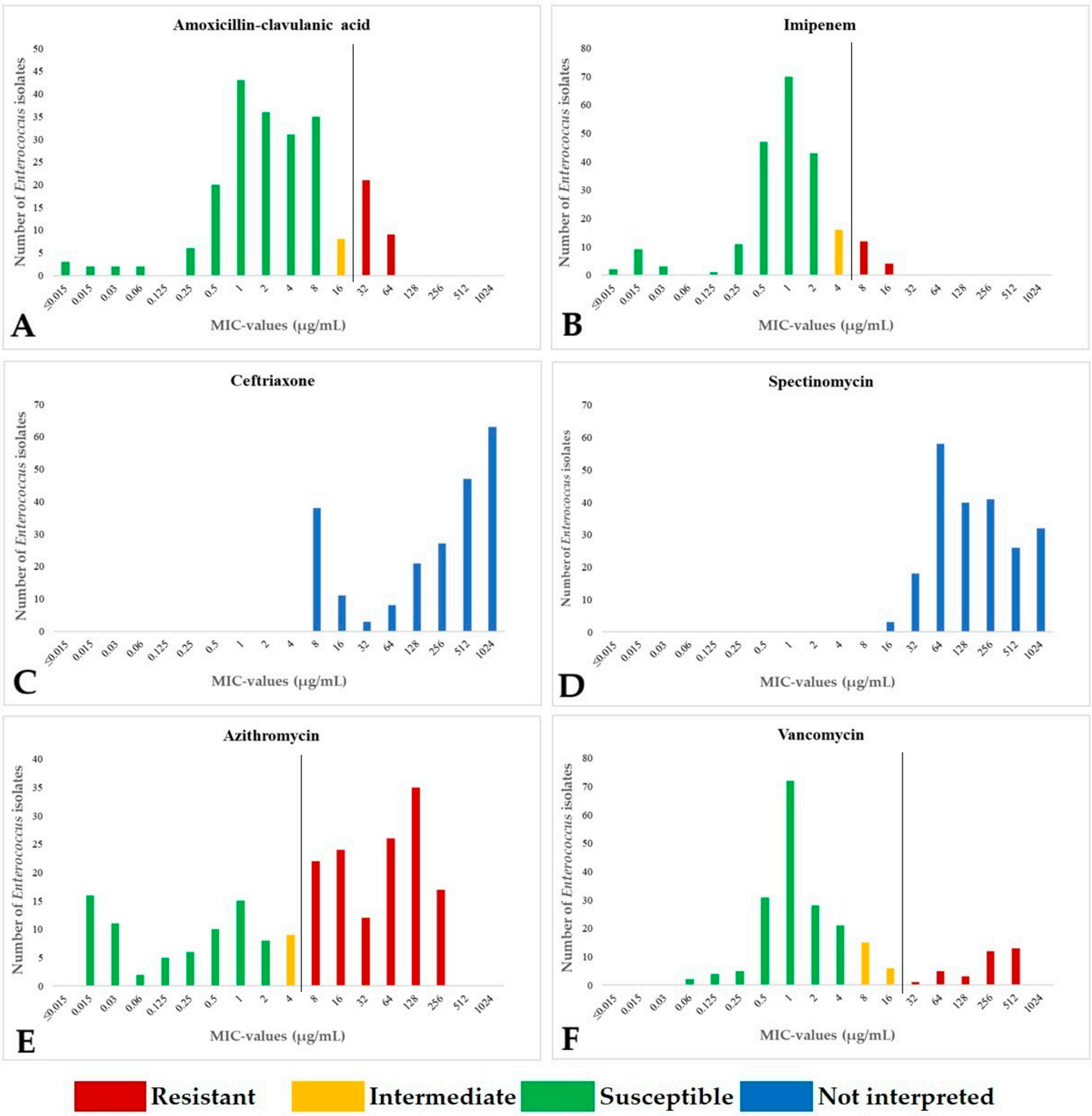

Supplementary Figure S3. Minimum inhibitory concentration (MIC) distributions of poultry-derived *Enterococcus* spp. isolates for antimicrobials primarily used in human medicine, including vancomycin. Bars represent the number of isolates at each broth microdilution concentration (two-fold dilution series; µg/mL). Colors denote Clinical Laboratory Standards Institute (CLSI) clinical categories (Susceptible/Intermediate/Resistant) where breakpoints are available; antimicrobials lacking clinical breakpoints are shown as Not interpreted (no clinical breakpoints available). Vertical dashed lines indicate clinical breakpoints. Panels: (A) amoxicillin–clavulanic acid, (B) imipenem, (C) ceftriaxone, (D) spectinomycin, (E) azithromycin, (F) vancomycin. Intermediate category is displayed only for antimicrobials with defined CLSI intermediate breakpoints.

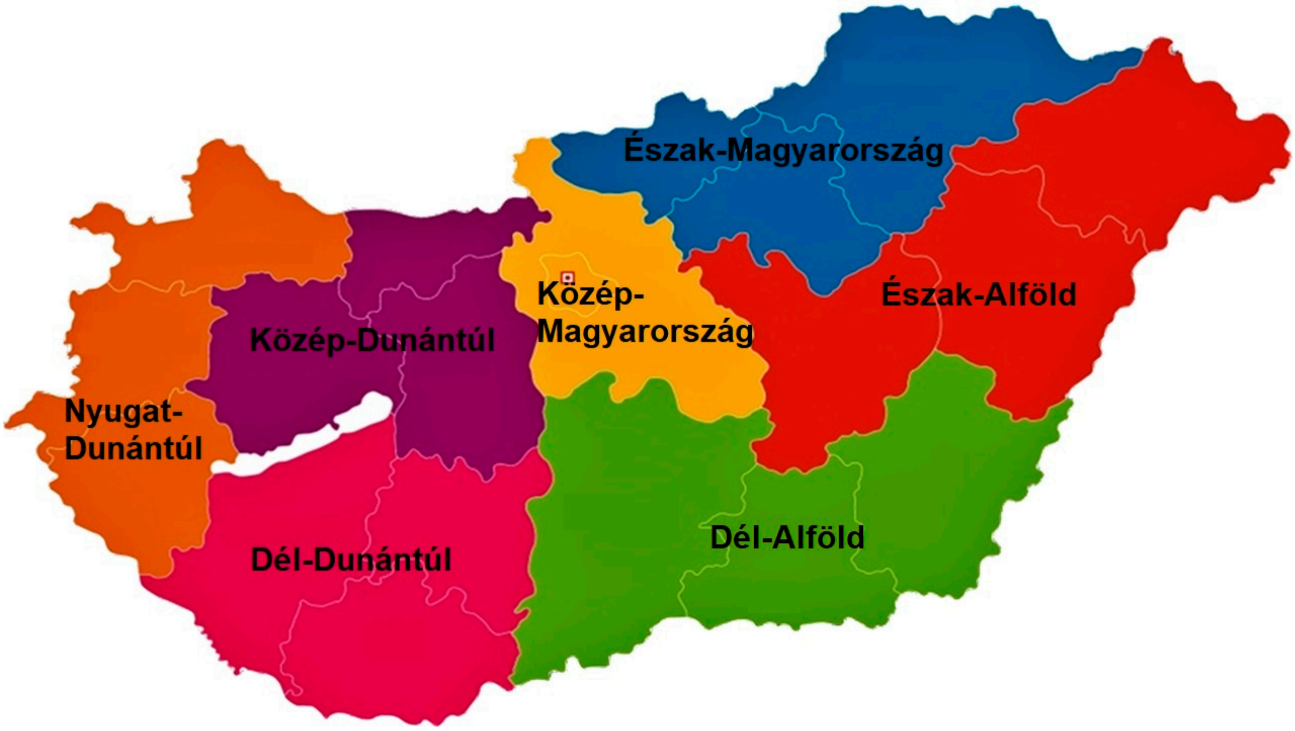

Supplementary Figure S4. Geographic outline of Hungary showing the seven NUTS-2 regions used for regional stratification in this study (Dél-Alföld, Észak-Magyarország, Nyugat-Dunántúl, Közép-Magyarország, Észak-Alföld, Dél-Dunántúl, and Közép-Dunántúl).
